# Primary Rhinoplasty

**Published:** 2013-01-15

**Authors:** Craig Pastor, Paul J. Therattil, Edward S. Lee

**Affiliations:** Department of Surgery, Division of Plastic Surgery, New Jersey Medical School, University of Medicine and Dentistry of New Jersey, Newark, NJ

**Figure:**
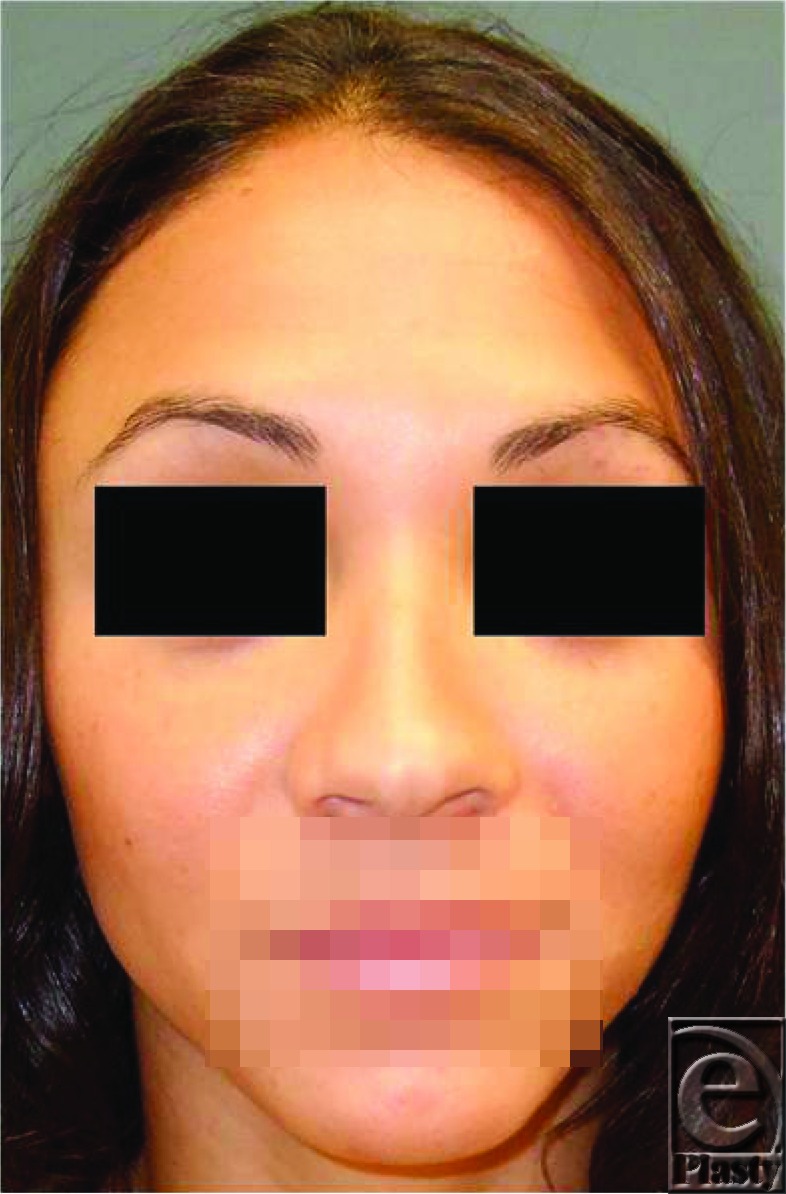


## DESCRIPTION

The patient is a 24-year-old woman who presents for a cosmetic rhinoplasty forimprovement of her wide alar base.

## QUESTIONS

1. **What are the preoperative considerations in rhinoplasty?**
2. **What are the different approaches to perform a rhinoplasty?**
3. **What are the different techniques available to shape the nasal tip?**
4. **Describe the effects of camouflage or the interplay of nasal shape.**

## DISCUSSION

Preoperative considerations in rhinoplasty include patient expectations, breathing issues, achieving aesthetic ideals, and chin position. In the initial consultation, understanding the patient's expectations is critical in attaining a successful outcome. Asking patients to rank their complaints in order of importance can help guide the surgeons approach, keeping in mind that patients who focus on minor or uncorrectable problems will likely be disappointed with even the best result. Whereas patients who present with breathing difficulties may benefit from a more functional rhinoplasty that focuses on remedying a deviated septum or obstructive hypertrophic inferior turbinates, many patients seek to achieve a nose that possesses aesthetic ideals. These ideals are derived from a series of classic facial measurements. For this reason, performing a systematic nasal analysis is critical when evaluating a patient for rhinoplasty. The nasal frontal angle height and depth; the bony pyramid; upper and lower lateral cartilages; nasal tip projection, rotation, symmetry, and position of tip-defining points; alar width, collapse, or retraction; and columellar show and angles must all be considered. In addition, a nasofacial analysis must also be performed. The lip-chin relationship is important and can be assessed on a lateral photograph.

Once the decision to perform a rhinoplasty has been made, the surgeon must determine the best operative approach, open versus closed. Each approach has its own advantages and disadvantages. The open approach allows for a more accurate anatomic diagnosis with undistorted exposure for consistent and reliable modification of nasal framework. In addition, with the open approach, there are more options with original tissues and grafts, and suture stabilization of grafts is possible. The closed approach avoids a columellar scar and allows for a potentially more rapid recovery due to less edema. This approach is ideal for patients requiring minimal tip work. Incisions in closed rhinoplasty include intracartilaginous or a combination ofinfracartilaginous and intercartilaginous (cartilage delivery technique).The intracartilaginous incision is directly into the lower lateral cartilage and can be used for cephalic resection, whereas the cartilage delivery technique delivers the entire lower lateral cartilage into the wound.

Multiple methods can be employed to reshape the nasal tip. These methods include suture techniques, cartilage grafts, performing a cephalic trim, and the use of a columellar strut. Suture techniques include the medial crural, interdomal, transdomal, and intercrural septal suture techniques. The medial crural suture stabilizes the tip complex. In cases where a columellar strut is used, the medial crural suture stabilizes it in place. Interdomal sutures achieve the goal of increased tip and columellar projection while transdomal sutures control dome symmetry. The intercrural septal suture and columellar strut also alter tip projection. Tip grafts are reserved for those cases when adequate projection, definition, or symmetry cannot be achieved with suture techniques alone. The cephalic trim procedure is indicated in cases of bulbous or boxy domes leading to paradomal fullness. The cephalic portion of the lower lateral cartilage is resected leaving at least a 5-mm strip.

The interplay between the different subunits of the nose can affect one's perception. For example, the appearance of a wide nasal base may be altered without altering the soft tissue at the base. By raising the nasal dorsum or nasofrontal angle, the appearance of an enlarged base is offset, because the nasal base appears smaller when the dorsum is higher. Another method to reduce a wide nostril base is the weir excision, which is a wedge excision of alar tissue.

Postoperative commentary: This patient underwent a cephalic trim procedure of her lower lateral cartilage with transdomal and intradomal sutures performed to improve her boxy nasal tip. Weir excisions were performed to reduce her alar flaring.

## Figures and Tables

**Figure 1:**
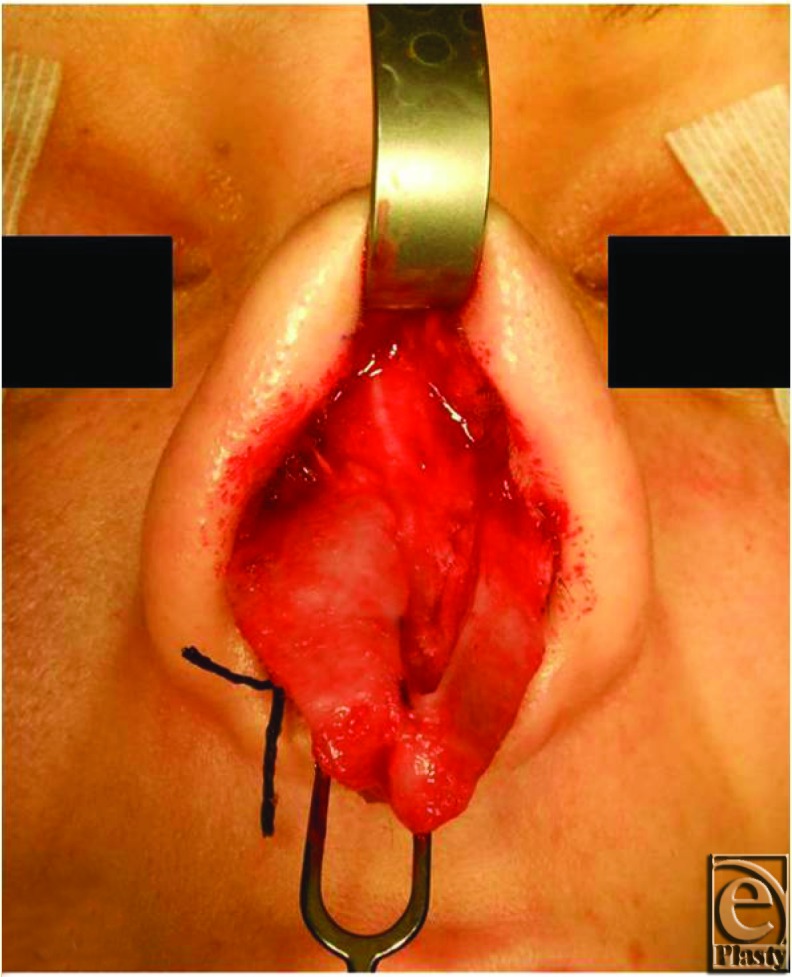
Following cephalic trim of the left lower lateral cartilage.

**Figure 2:**
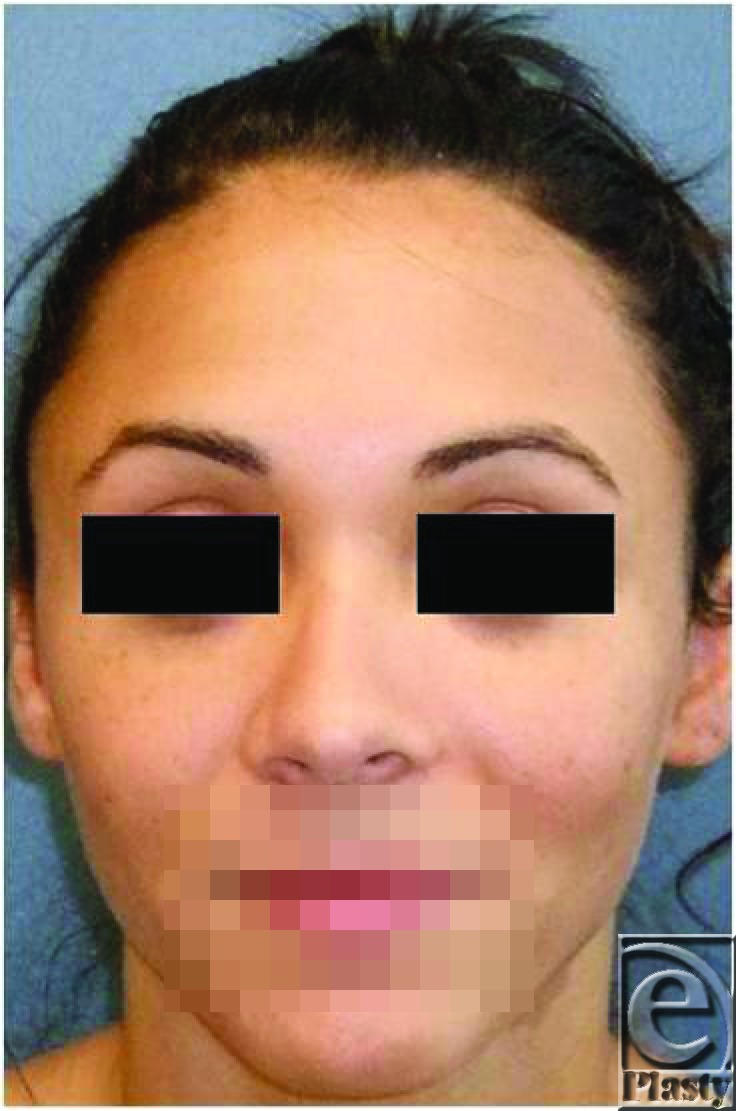
Status-post cephalic trim, transdomal and intradomal sutures, and Weirexcisions.
